# Considering depression as a secondary outcome in the optimization of physical activity interventions for breast cancer survivors in the PACES trial: a factorial randomized controlled trial

**DOI:** 10.1186/s12966-023-01437-x

**Published:** 2023-04-20

**Authors:** Chad D. Rethorst, Thomas J. Carmody, Keith E. Argenbright, Taryn L. Mayes, Heidi A. Hamann, Madhukar H. Trivedi

**Affiliations:** 1 Institute for Advancing Health Through Agriculture, Texas A&M Agrilife Research, 17360 Coit Road, Dallas, TX 75252 USA; 2 Department of Population and Data Sciences, University of Texas Southwestern Medical Center, Dallas, TX USA; 3 Moncrief Cancer Institute, University of Texas Southwestern Medical Center, Dallas, TX Fort Worth, TX USA; 4 Harold C. Simmons Cancer Center, University of Texas Southwestern Medical Center, Dallas, TX USA; 5 Department of Psychiatry, University of Texas Southwestern Medical Center, Dallas, TX USA; 6 Department of Psychology, University of Arizona, Tucson, AZ USA

**Keywords:** Physical activity, Depression, Breast cancer survivors, Intervention optimization, Behavioral interventions

## Abstract

**Background:**

Depressive symptoms result in considerable burden for breast cancer survivors. Increased physical activity may reduce these burdens but existing evidence from physical activity interventions in equivocal. Furthermore, physical activity intervention strategies may differentially impact depressive symptoms, which should be considered in designing and optimizing behavioral interventions for breast cancer survivors.

**Methods:**

The Physical Activity for Cancer Survivors (PACES) trial enrolled 336 participants breast cancer survivors, who were 3 months to 10 years post-treatment, and insufficiently active (< 150 min of moderate-to-vigorous physical activity per week). Participants were randomly assigned to a combination of 4 intervention strategies in a full-factorial design: 1) supervised exercise sessions, 2) facility access, 3) Active Living Every Day, and 4) Fitbit self-monitoring. Depressive symptoms were assessed at baseline, mid-intervention (3 months), and post-intervention (6 months) using the Quick Inventory for Depressive Symptoms. Change in depressive symptoms were analyzed using a linear mixed-effects model.

**Results:**

Results from the linear mixed-effects model indicated that depressive symptoms decreased significantly across the entire study sample over the 6-month intervention (F = 4.09, *p* = 0.044). A significant ALED x time interaction indicated participants who received the ALED intervention experienced greater reductions in depressive symptoms (F = 5.29, *p* = 0.022). No other intervention strategy significantly impacted depressive symptoms.

**Conclusions:**

The ALED intervention consists of strategies (i.e., goal setting, social support) that may have a beneficial impact on depressive symptoms above and beyond the effect of increased physical activity. Our findings highlight the need to consider secondary outcomes when designing and optimizing physical activity interventions.

**Trial registration:**

ClinicalTrials.gov NCT03060941. Posted February 23, 2017.

## Introduction

Breast cancer is the most common cancer in women and a significant cause of mortality and morbidity in the United States [1]. Advances in breast cancer diagnosis and treatment have improved survival rates and prolonged life spans, thus increasing the number of breast cancer survivors, with 3.8 million breast cancer survivors in 2019 and an estimated 4.9 million survivors by 2030 [2]. Following treatment, breast cancer survivors report ongoing psychological symptoms such as depression and depressive symptoms. A recent meta-analysis estimated the global prevalence of depression among breast cancer survivors to be 32.2%, [3] Depression among breast cancer survivors negatively affects quality of life, increases risk of disease recurrence and all-cause mortality [4–9]. Given the growing number of breast cancer survivors and the negative impact of depression on long-term health for breast cancer survivors, there is a dire need to develop evidence-based programs and services to improve their psychological and physical well-being.

Exercise has the potential to improve psychological and physical health during breast cancer survivorship. The American Cancer Society and the American College of Sports Medicine both recommend that all breast cancer survivors engage in regular physical activity [1, 10] as physical activity has been shown to be associated with a decreased risk of recurrence [11], while enhancing overall quality of life measures in breast cancer survivors [12–14]. It has long been established that physical activity can help in the treatment and prevention of depression and depressive symptoms [15–17]. Randomized controlled trials of structured exercise interventions among breast cancer survivors have yielded significant reductions in depressive symptoms [18, 19].

Of note, these trials implemented structured exercise interventions.While there is substantial evidence supporting behavioral interventions for increasing physical activity among breast cancer survivors, [20] few trials have reported depressive symptom outcomes and evidence from these trials is mixed. Rogers et al. [21] report a significant reduction in depressive symptoms following a 3-month physical activity intervention but note that prior studies [22, 23] of physical activity interventions did not yield significant reductions in depressive symptoms.

Physical activity interventions target factors such as increased social interaction, social support, and self-efficacy, all of which have all been shown to have positive effects on depression [24, 25]. Therefore, it is possible that physical activity interventions might result in improvements in depressive symptoms that are not attributable to increase physical activity, and that physical activity intervention strategies might differ in their effect on depressive symptoms. Such findings would have implications for the optimization of physical activity interventions for breast cancer survivors. As noted by Collins et al. [26, 27], the Multiphase Optimization Strategy (MOST) aims to optimize a behavioral intervention relative to an a priori primary outcome. However, behavioral interventions often target a behavior as a proximal outcome under the rationale that improving the proximal behavior will exert an effect on a more distal health outcome [28]. In the example of physical activity, it is presumed that increasing physical activity will improve multiple distal outcomes (i.e., disease recurrence, depressive symptoms, quality of life, mortality). However, as outlined above, it is possible that in the case of depressive symptoms, behavioral strategies may differ in their effects. As such, exploring those effect would inform future optimization efforts that might consider different intervention strategies for sub-populations (i.e., breast cancer survivors with depression vs. breast cancer survivors without depression).

The Promoting Activity in Cancer Survivors (PACES) trial provides an opportunity to address these questions. The PACES trial implemented a factorial design, as suggested under the MOST framework [26, 27], to evaluate four intervention strategies for increasing physical activity among breast cancer survivors [29]. While the primary goal of PACES is to optimize an intervention to increase physical activity, selection of intervention components should not only consider physical activity but other outcomes, like depression, that can improve well-being and ultimately health outcomes of breast cancer survivors. The current analysis will evaluate the effects of each intervention strategy on depressive symptoms.

## Methods

### Study and intervention overview

Full methodological details for the PACES trial have been previously published [29]. Details relevant to the reported analysis are described below. The study protocol was approved by the UT Southwestern IRB and registered with ClinicalTrials.gov (NCT03060941). Participants signed an informed consent document prior to completing any study activities. Outcomes were assessed at three timepoints: baseline, Week 13, and Week 25. Visits were conducted at the UT Southwestern Center for Depression Research and Clinical Care (Dallas, TX) and the Moncrief Cancer Institute (Fort Worth, TX). Data was collected December 2016 to February 2020.

### Participants

Participants were recruited via emails, flyers posted in clinics, and social media postings. Individuals were eligible for participation if they were: 1) women diagnosed with breast cancer (stages 1–4), 2) completed treatment > 3 months and < 5 years prior to enrollment, 3) engage in < 150 min of moderate-to-vigorous physical activity (MVPA) per week as assessed by the International Physical Activity Questionnaire, 4) able to engage in physical activity. To ensure recruitment of the target sample size, we expanded inclusion to breast cancer survivors up to 10 years post-treatment during the trial in July 2017 (162 participants were enrolled prior to the change in study eligibility). Exclusion criteria were: 1) a medical condition contraindicating physical activity participation, or 2) unable/unwilling to provide informed consent.

### Intervention

In the full factorial design, participants were randomized to receive a combination of up to four intervention components (Table 1). The randomization sequence was generated by the study statistician, was stratified by clinical treatment site, with balanced blocks which varied in size through random permutation. In addition, all participants received a book, “Exercise for Health: An Exercise Guide for Breast Cancer Survivors,” with demonstrated efficacy for increasing physical activity among breast cancer survivors [30, 31]. Intervention components were delivered concurrently; participants assigned to more than one intervention components engaged in those components as described.

**Table 1:** PACES intervention components

	**Group 1**	**Group 2**	**Group 3**	**Group 4**	**Group 5**	**Group 6**	**Group 7**	**Group 8**	**Group 9**	**Group 10**	**Group 11**	**Group 12**	**Group 13**	**Group 14**	**Group 15**	**Group 16**
PA Education	X	X	X	X	X	X	X	X	X	X	X	X	X	X	X	X
ALED		X				X	X	X				X	X	X		X
Facility Access			X			X			X	X		X		X	X	X
Fitbit				X			X		X		X	X	X		X	X
Supervised Exercise					X			X		X	X		X	X	X	X

#### Supervised exercise sessions

Participants were given a goal of completing 150 min of weekly MVPA through a combination of supervised, in-person sessions at either UT Southwestern or Moncrief Cancer Institute and unsupervised sessions completed during the first 6 weeks of the study period. The number of weekly sessions was tapered over the 6 weeks to facilitate transition to completing unsupervised sessions. In the first two weeks of the intervention, participants completed 3 supervised sessions on either a treadmill or stationary bicycle and completed at least one unsupervised session per week. Over the next four weeks, the number of supervised sessions decreased (two supervised sessions in Weeks 3–4, one supervised session in Weeks 5–6) and unsupervised sessions increased to achieve the 150 min/week goal. Sessions were supervised in a one-on-one setting by a trained interventionist.

#### Facility access

Participants received a six-month membership to a local fitness facility. Study staff worked with participants to find a convenient facility for each participant.

#### Active Living Every Day (ALED)

The ALED program has proven effective in increasing physical activity across several populations, including breast cancer survivors [32–34]. Participants attended 12 bi-weekly education sessions, led by a trained interventionist at either UT Southwestern or Moncrief Cancer Institute, that aimed to supporting behavioral strategies for increasing physical activity, led by a trained interventionist.

#### Fitbit

Participants were provided a Fitbit Alta HR for 24 weeks and were provided a one-page information sheet on effective goal-setting and self-monitoring strategies. Smartphone ownership was not required for participation. For participants with a smartphone, the Fitbit mobile application was installed on their smartphone. In the event a participant did not own a smartphone, they were provided instructions for syncing their Fitbit on a personal computer and were also provided a paper log that they could use to track their activity.

### Measures

Demographic information, height, and weight were collected at baseline. Depressive symptoms were measured via the self-rated Quick Inventory of Depressive Symptomatology (QIDS-SR) [35] at baseline, Week 13, and Week 25.

### Statistical analysis

A linear mixed-effects model with random participant effects and fixed time effects was fit for QIDS-SR total score. The model contained terms for baseline covariates (age, ethnicity, race, and BMI), assessment time (3 months and 6 months), and four categorical variables indicating each exercise intervention’s inclusion or exclusion status. Two-way interaction terms across all interventions and time were included. Sub-sample analyses were conducted in participants with significant depressive symptoms at baselines, defined as a QIDS-SR score of ≥ 5 [35, 36].

## Results

**Fig. 1:**
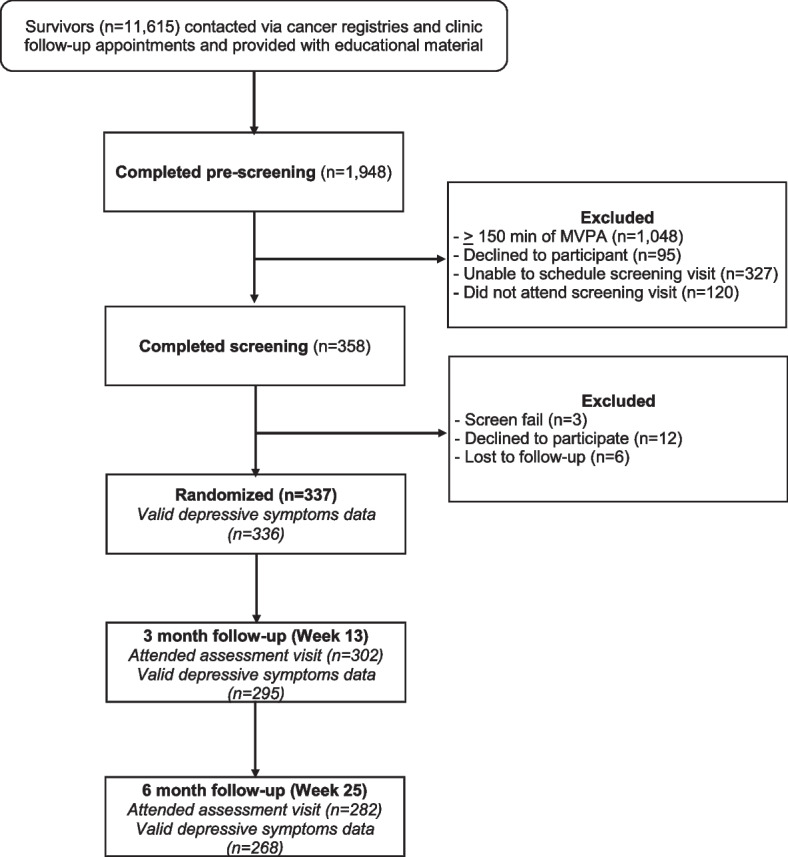
CONSORT diagram

Three hundred thirty seven individuals attended the baseline evaluation and were randomized (Fig. 1); 336 participants provided QIDS-SR data at baseline. Relevant baseline characteristics are presented in Table 2 and QIDS-SR means by visit and treatment group are presented in Table 3.

**Table 2:** Baseline characteristics

Variable	n	Mean (SD)
Age (yrs)	337	57.63 (11)
Race		
Black (%)	48	14.77%
White (%)	251	77.23%
Other (%)	26	8.00%
Hispanic Ethnicity (%)	36	10.78%
Depressive Symptoms (QIDS-SR)	336	6.66 (3.8)

**Table 3:** Depressive symptoms (QIDS-SR score) by time and intervention assignment

Group	Time	n	Mean (SD)
All	Baseline	336	6.66 (3.8)
	Week 13	295	5.34 (3.2)
	Week 25	268	5.03 (3.1)
ALED	Baseline	169	6.62 (3.6)
	Week 13	145	5.35 (3.4)
	Week 25	131	4.67 (3.0)
Facility Access	Baseline	168	6.55 (4.0)
	Week 13	151	5.24 (3.3)
	Week 25	137	5.14 (3.4)
Fitbit	Baseline	166	6.73 (3.8)
	Week 13	152	5.26 (3.2)
	Week 25	139	4.98 (2.7)
Supervised Exercise	Baseline	170	6.32 (3.8)
	Week 13	152	5.07 (3.2)
	Week 25	136	4.82 (3.0)

### Intervention effects on depressive symptoms

Results of the mixed model analysis indicated improvement in depressive symptoms over time when averaged across all study interventions (F = 4.09, *p* = 0.044). As shown in Table 4, the analysis revealed a significant time x ALED interaction (F = 5.29, *p* = 0.022) indicating participants assigned to the ALED condition experienced greater reductions in depressive symptoms. Time interaction effects were not significant for supervised exercise sessions (*p* = 0.301), facility access (*p* = 0.193), or Fitbit (*p* = 0.620).

**Table 4:** Fixed effects of intervention x time on depressive symptoms (QIDS-SR score)

	**Estimate**	**SE**	***F***	***p* value**
ALED	-0.72	0.3	5.29	0.022
Facility Access	0.323	0.3	1.07	0.301
Fitbit	0.409	0.3	1.7	0.193
Supervised Exercise	0.155	0.3	0.25	0.62

In the subsample analysis of participants with significant depressive symptoms (*n* = 167), (Table 5), the overall effect of time was not significant (F = 1.16, *p* = 0.283). Consistent with the full sample analysis, the time x ALED interaction was significant (F = 7.01, *p* = 0. 009). Time x intervention effects of the three other interventions were not significant.

**Table 5:** Fixed effects of intervention x time on depressive symptoms (QIDS-SR score) in participants with elevated depressive symptoms at baseline

	**Estimate**	**SE**	***F***	***p* value**
ALED	-1.22	0.5	7.01	0.009
Facility Access	0.452	0.5	0.95	0.331
Fitbit	-0.231	0.5	0.25	0.621
Supervised Exercise	0.403	0.5	0.75	0.389

## Discussion

Depression is a common problem among breast cancer survivors that results in significant burden and has implication for long-term survival. Behavioral interventions that target an increase in physical activity may have a role in reducing depressive symptoms among breast cancer survivors. Results of the current analysis indicate that depressive symptoms were reduced across a sample of over 300 breast cancer survivors. Through the factorial design, we identified one specific behavioral intervention strategy, ALED, that resulted in significant reductions in depressive symptoms.

Results of the current analysis are in contrast to findings of the primary outcoming analysis (under review). In that analysis, participants who engaged in the supervised exercise sessions demonstrated greater engagement and MVPA at six months compared to those participants who did not receive supervised exercise. No other intervention strategy significantly increased MVPA. However, participants engaged in the ALED intervention engaged in less light physical activity. Those findings, combined with the fact that change in physical activity was not correlated with changing depressive symptoms suggest that characteristics of the ALED intervention beyond physical activity engagement might be responsible for the change in depressive symptoms observed.

ALED is behavior change program designed around skill development in goal-setting, identifying and addressing barriers, and building social support. While within ALED these skills are targeting changes in physical activity behavior, it is possible that the development of these skills has application in other aspects of life that could positively impact depressive symptoms. For example, it has been postulated that goal-setting and goal pursuit may improve depressive symptoms [24]. Similarly, larger social network size and greater perceived social support have been associated with improved treatment outcomes for persons with depression [25]. ALED is delivered in a group setting that may further facilitate development of social support. As such, it is unsurprising that ALED may have effects on depressive symptoms above and beyond any effects resulting from an increase in physical activity. Previous research also supports the potential for ALED to improve depressive symptoms. In a previous study that evaluated the dissemination of ALED in community settings, older adults participating in ALED experienced significant decreases in depressive symptoms [37].

It should be noted that our findings should not suggest that increasing physical activity cannot improve depressive symptoms among breast cancer survivors. In fact, the overall decrease in depressive symptoms observed across the entire study population would suggest increasing physical activity can improve depressive symptoms among breast cancer survivors. Instead, our findings have potentially important implications in the aim to optimize behavioral interventions for breast cancer survivors. In the PACES trial, the primary outcome was MVPA and our primary outcome analysis found that supervised exercise sessions were the only intervention strategy that significantly increased MVPA. However, supervised sessions did not significantly improve depressive symptoms in the current analysis. These findings suggest that multiple outcomes might be considered when determining an “optimal” intervention for a population and that a more personalized approach might be warranted.

The results presented should be interpreted with consideration of the strengths and limitations of the study. The factorial design allowed for concurrent evaluation of the effect of multiple intervention components on depressive symptoms. However, the design does not provide a true “control” condition; therefore, it is possible that some of the improvement in depressive symptoms observed was due to factors beyond intervention participation. We note that depressive symptoms was a secondary outcome. However, the large sample size provided the necessary power to detect a significant effect of ALED. The focus of this paper was on depressive symptoms; however, multiple outcomes and individual factors could ultimately inform what is the “optimal” intervention for a particular group, subgroup, or individual.

Our findings have both scientific and practical implications. Scientifically, the results highlight the need for future research identifying factors to inform intervention optimization. Ultimately our findings reinforce the concept of intervention optimization as an iterative process. As noted by Collins et al. [26, 27], the optimization phase of the MOST process can lead to further refinement and optimization studies. Our findings would suggest that further optimization specific to breast cancer survivors might be warranted. For example, for breast cancer survivors with depression, a multi-component intervention that included both supervised exercise sessions and ALED might be most effective in improving long-term outcomes for breast cancer survivors with depression. Practically, this highlights the need for frameworks that guide researchers and decision makers on how to integrate multiple factors to inform intervention optimization.

## Conclusion

Our results would suggest that ALED specifically improves depressive symptoms significantly, while no other intervention strategy significantly affected depressive symptoms. When combined with our prior findings, it would suggest that multiple outcomes might be considered in determining the “optimal” intervention approach for a population. In fact, our findings would suggest that a more individual approach might be warranted to best match the desired outcomes most relevant for a particular individual.

## Data Availability

The datasets used and/or analysed during the current study are available from the corresponding author on reasonable request.

## Abbreviations

ALED: Active Living Every Day
MOST: Multiphase Optimization Strategy
MVPA: Moderate-to-vigorous physical activity
QIDS-SR: Quick Inventory of Depressive Symptoms – Self-Report
